# Frequency of rhinitis and orofacial disorders in patients with dental malocclusion

**DOI:** 10.1016/j.rppede.2016.02.009

**Published:** 2016

**Authors:** Tamara Christine de Souza Imbaud, Márcia Carvalho Mallozi, Vanda Beatriz Teixeira Coelho Domingos, Dirceu Solé

**Affiliations:** Escola Paulista de Medicina, Universidade Federal de São Paulo (EPM-Unifesp), São Paulo, SP, Brazil

**Keywords:** Rhinitis, Oral breathing, Malocclusion, Cephalometry, Bruxism

## Abstract

**Objective:**

To describe the frequency and etiology of rhinitis, oral breathing, types of malocclusion and orofacial disorders in patients treated for dental malocclusion.

**Methods:**

Patients with poor dental occlusion (*n*=89, 8-15 years) undergoing orthodontic treatment at the Postgraduate Orthodontics Center (São Paulo, Brazil) participated in the study. Rhinitis and oral breathing were diagnosed by anamnesis, clinical assessment and allergic etiology of rhinitis through immediate hypersensitivity skin prick test with airborne allergens. The association between types of breathing (oral or nasal), rhinitis and types of dental malocclusion, bruxism and cephalometric alterations (increased *Y* axis of facial growth) compared to standard cephalometric tracing (Escola de Odontologia da Universidade de São Paulo) were assessed.

**Results:**

The frequency of rhinitis in patients with dental malocclusion was 76.4% (68), and, of these, 81.7% were allergic (49/60 positive skin prick test), whereas the frequency of oral breathing was 62.9%. There was a significant association between an increased *Y* axis of facial growth and oral breathing (*p*<0.001), as well as between oral breathing and rhinitis (*p*=0.009). There was no association between rhinitis and bruxism.

**Conclusions:**

The frequency of rhinitis in children with dental malocclusion is higher than that in the general population, which is approximately 30%. Patients with oral breathing have a tendency to a dolichofacial growth pattern (increased *Y* axis of facial growth). In patients with rhinitis, regardless of the presence of oral breathing, the dolichofacial growth tendency was not observed.

## Resumo

**Objetivo:**

Descrever a frequência e etiologia da rinite, da respiração oral, os tipos de má oclusão e as alterações orofaciais em pacientes tratados por má oclusão dentária.

**Métodos:**

Pacientes com má oclusão dentária (*n*=89, oito a 15 anos) em tratamento ortodôntico em centro de pós-graduação em ortodontia (São Paulo, Brasil) participaram do estudo. Rinite e respiração oral foram diagnosticadas por anamnese e exame clínico e a etiologia alérgica dessa por teste cutâneo de hipersensibilidade imediata (TCHI) com aeroalérgenos. Avaliou-se a relação entre tipos de respiração (oral ou nasal), rinite e tipos de má oclusão dentária, bruxismo e alterações cefalométricas (aumento do eixo Y de crescimento facial) em comparação com o traçado cefalométrico padrão (Escola de Odontologia da Universidade de São Paulo).

**Resultados:**

A frequência de rinite nos pacientes com má oclusão dentária foi de 76,4% (68), desses 81,7% eram alérgicos (49/60 TCHI positivo) e a frequência de respiração oral foi de 62,9%. Houve associação significativa entre ter o eixo Y de crescimento facial aumentado e respiração oral (*p*<0,001), o mesmo entre respiração oral e rinite (*p*=0,009). Não houve associação entre rinite e bruxismo.

**Conclusões:**

A frequência de rinite em crianças com má oclusão dentária é superior à da população geral, que gira ao redor de 30%. Os pacientes com respiração oral têm tendência de crescimento dólico facial (eixo Y de crescimento aumentado). Nos pacientes com rinite, independentemente da presença da respiração oral, a tendência dólico facial não foi observada.

## Introduction

The growth and development of the craniofacial structure and, consequently, the dental occlusion, undergo environmental influences through breathing, breastfeeding, chewing, habits (use of bottle and digit and/or pacifier sucking) and swallowing.^1^^,^ 2

Through the aeration of the pneumatic paranasal sinuses, breathing allows adequate facial development through pressure from the air flow and backflow through the nostrils. Obstruction in the airways, such as adenoid and tonsil hypertrophy, interferes with the inspiratory pressure. The scarce nasal flow and the absence of tongue pressure against the palate lead to maxillary sinus hypoplasia, the narrowing of the nasal cavities and the upper dental arch, which favors dental malocclusion.^3^^-^ 5 Mouth breathing can be favored by the delay in the diagnosis and treatment of allergic rhinitis (AR), which, in addition to facilitating chronic mouth breathing, can result in speech disorder, chronic sinusitis, bruxism, nocturnal apnea, sleep disorders, auditory tube dysfunction, otitis media and asthma attacks.^6^ Adenoid and tonsil hypertrophy and posterior cross-bite are associated with otitis media in children.^2^^,^ 7 ^,^ 8

AR is considered a public health problem due to its high prevalence, as it impairs patient quality of life and has high social cost.^6^^,^ 10 The prevalence of AR in Brazilian schoolchildren varies between 26.6% and 34.2%.^11^ Although the association between dental malocclusion and AR is common, their interrelationships deserve further study. The association between dental malocclusion and oral breathing in patients with AR,^12^^-^ 15 as well as bruxism,^13^ has been reported.

Reduction of craniofacial diameters, dental malocclusion (anterior dental crowding, cross-bite, protruding jaw, receding jaw) and direction of facial growth vector with a predominance of the vertical component, which is expressed by an increase in the growth *Y* axis in the cephalometric analysis have been described in patients with AR.^1^^,^ 12 ^-^ 16 Dental malocclusion is associated with other disorders, such as mouth breathing, use of pacifier and thumb/digit sucking for a long time (after three and four years of age, respectively).^2^^,^ 12 ^-^ 23 A study of children aged 5-6 years enrolled in elementary schools in Brazil showed high frequency of malocclusion, which was associated with oral habits such as the use of pacifier, bottle-feeding and thumb/digit sucking.^1^^,^ 12 Therefore, health professionals, doctors, dentists and speech therapists should be more aware of the negative impact of airway obstruction on the patient's facial growth and of their psychological health.^2^^,^ 13

The multidisciplinary evaluation of patients with rhinitis and/or mouth breathing treated for dental malocclusion is important for a more appropriate management.^2^ In this study, we evaluated patients undergoing treatment for dental malocclusion at the Orthodontics Service regarding the frequency of rhinitis, mouth breathing, bruxism and orofacial alterations, as well as the increase in the *Y* axis through cephalometric evaluation, according to the presence or absence of rhinitis and/or mouth breathing.

## Method

A total of 89 patients were selected (8-15 years of age) among those treated at the service (300 patients older than seven years) in a center specializing in orthodontics in São Paulo, referred for orthodontic treatment for dental malocclusion, during 2012. The choice of patients was made at random and those reporting habits such as pacifier use or thumb/digit sucking for a period longer than three and four years, respectively, were excluded, as well as those diagnosed with adenoid tonsil hypertrophy (X-ray) or surgery (adenoidectomy), osteo-dental discrepancy, abnormal nasal pyramid that could interfere with nasal breathing, atypical deglutition and genetic malformations. All patients underwent swallowing evaluation by a speech therapist before starting treatment. All patients had the authorization of their parents/tutors to participate and the latter signed the informed consent form. Patients were assessed through clinical history and clinical examination, with special attention to the oral cavity and nasal passages to attain the diagnosis of rhinitis and/or mouth breathing (TCSI). Mouth breathers were considered as those patients whose breathing was predominantly through the mouth over the last six months^24^ (*n*=56), with the others being characterized as nasal breathers (*n*=33).

Patients that showed nasal signs and symptoms such as sneezing, runny nose, nasal obstruction and/or nasal itching were identified as having rhinitis.^6^ According to this criterion, patients were divided into two groups: those with rhinitis (*n*=68) and without rhinitis (*n*=21).

All patients with rhinitis were submitted to the skin prick test (SPT)^25^ by same investigator (TCSI) to identify the etiology. The skin prick technique was used with the standard battery of aeroallergens (*Dermatophagoides pteronyssinus*, *Dermatophagoides farinae*, *Blomia tropicalis*, fungal mix, pollen mix, *Blattella germanica*, dog epithelium, cat epithelium, histamine (1mg/mL) and negative control - FDA Allergen^®^). The appearance of papules with a mean diameter of 3mm larger than the diameter of the negative control to any aeroallergen characterized the SPT as positive and the patient as having AR.^25^

**Figure 1:**
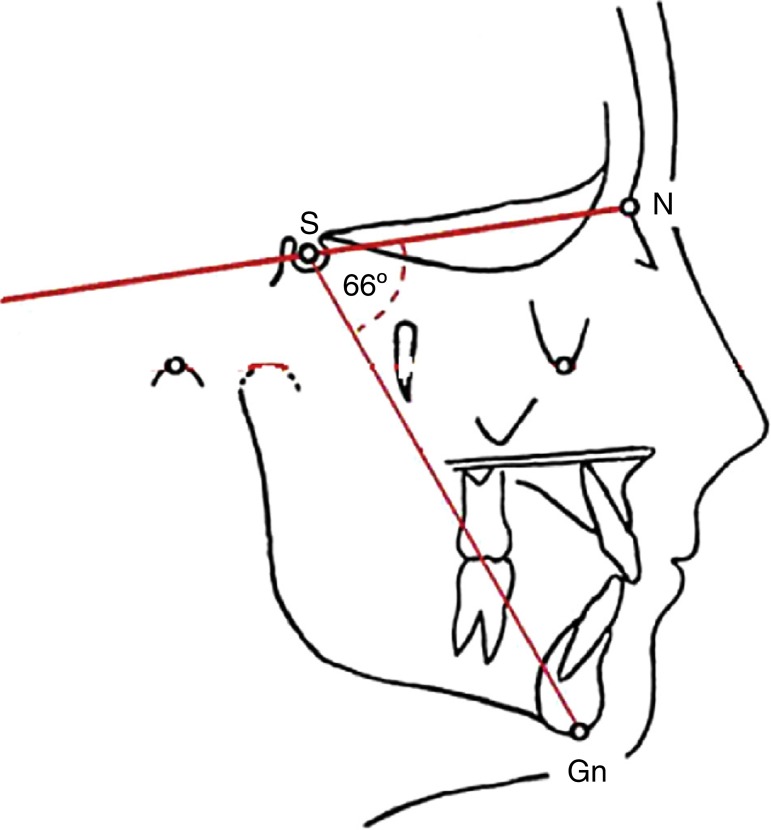
NS. Gn angle - *Y*-growth axis.

Patients (divided into groups, with or without rhinitis and with and without mouth breathing) were also evaluated for the presence of bruxism, type of malocclusion and increased *Y* axis. The *Y* axis (NS. Gn. angle, Fig. 1) was obtained through cephalometric assessment (VBTCD) made on the patient's radiography. The graphic representation of mandibular growth direction was made in relation to the base of the skull (USP standard).^26^ When increased, it indicates that the jaw grows clockwise, results in a longer face and retrognathia. When the *Y*-axis is decreased, it means that the growth occurs in a counterclockwise direction, which results in mandibular prognathism. These angular and linear measurements of facial, skeletal and dental characteristics were compared with the normal standards.^26^

The diagnosis of the type of malocclusion was made by an orthodontist (VBTCD) and the diagnosis of bruxism was based on information from parents about the habit of their children of grinding or clenching their teeth.

According to the analyzed variables, the following tests were employed: Student's *t* test, Fisher's exact and chi-square test and the level of rejection of the null hypothesis was set at 5%.

The study was approved by the Institutional Review Board of Hospital São Paulo, Escola Paulista de Medicina, Universidade Federal de São Paulo.

## Results

Rhinitis was diagnosed in 76.4% (68/89) of patients, with no significant differences regarding the median age: 144 months (96-180 months) for those with rhinitis and 120 months (90-180 months) for those without it.

Table 1 shows the alterations observed in patients according to the presence or absence of rhinitis. It also shows that the presence of mouth breathing was significantly more frequent in patients with rhinitis.

**Table 1:** Patients according to observed maxillofacial and occlusive alterations, considering the presence or absence of rhinitis or mouth breathing.

	Rhinitis				Mouth breathing		
	Yes (*n*=68)	No (*n*=21)	*OR* (95%CI)		Yes (*n*=56)	No (*n*=33)	*OR* (95%CI)
Maxillary atresia	29 (78.4)	8 (21.6)	1.21 (0.44-3.30)		24 (64.9)	13 (35.1)	1.15 (0.48-2.77)
Bruxism	28 (87.5)	4 (12.5)	2.98 (0.90-9.80)		23 (71.9)	9 (28.1)	1.86 (0.73-4.73)
Mouth breathing	48 (85.8)	8 (14.2)	***3.90*** a ***(1.40-0.86)***		-	-	-
Rhinitis	-	-	-		48 (70.5)	20 (29.5)	***3.91*** a ***(1.40-10.86)***
Increased *Y* axis	35 (81.3)	8 (16.7)	1.72 (0.63-4.69)		30 (85.7)	5 (14.3)	***6.46*** a ***(2.18-19.16)***
Deep bite	32 (74.4)	11 (25.6)	0.80 (0.30-2.15)		25 (58.1)	18 (41.9)	0.67 (0.28-1.60)
Open bite	24 (82.8)	5 (17.2)	1.75 (0.57-5.35)		13 (76.5)	4 (23.5)	2.19 (0.65-7.39)
Cross-bite	24 (82.8)	5 (17.2)	1.75 (0.57-5.35)		19 (65.5)	10 (34.5)	1.18 (0.47-2.98)
Dental crowding	41 (78.8)	11 (21.2)	1.38 (0.52-3.70)		35 (67.3)	17 (32.7)	1.60 (0.66-3.75)

*OR*, odds ratio; 95%CI, 95% confidence interval; Bold and italics, significant values *p*<0.05.

a Fisher's exact test.

Table 1 also shows the alterations observed in patients according to the presence (64.9%) or absence of mouth breathing. The presence of rhinitis and the increase in the *Y*-axis were significantly associated with oral breathing.

The frequency of allergic sensitization was 81.7%, significantly higher among patients with moderate/severe AR, when compared to those with less severe forms. *B. tropicalis* (41/49), *D. pteronyssinus* (40/49), *D. farinae* (40/49), *B. germanica* (6/49) and a fungal mix (5/49) were the identified allergens.

## Discussion

The association between oral breathing and rhinitis has been widely documented and occurs as a result of nasal obstruction, which is one of the most uncomfortable symptoms of rhinitis.^2^^,^ 6 ^,^ 11 ^,^ 14 Long-term studies with these patients have shown a higher frequency of facial development alterations and dental malocclusion, especially as a possible consequence of chronic mouth breathing.^2^^,^ 12 ^-^ 14 ^,^ 19 ^-^ 22 However, there have been few studies assessing the prevalence of rhinitis in patients with dental malocclusion, which motivated this investigation. As we realize the importance of breathing for orofacial development and dental occlusion,^1^^,^ 3 ^,^ 12 ^,^ 13 ^,^ 17 patients with other causes of mouth breathing were excluded to avoid interference with the results.^2^^-^ 4 ^,^ 27 ^,^ 28

Approximately 75% of the patients were diagnosed with rhinitis. This result far exceeds the values observed in epidemiological studies in the general population.^11^ Additionally, 81.7% of patients submitted to the SPT were diagnosed as sensitive to at least one aeroallergen, characterizing them as having AR. Similarly to what was reported by other authors, the presence of rhinitis was associated with mouth breathing,^6^^,^ 9 ^,^ 10 which did not occur with the other parameters (Table 1).

When analyzing the patients based on the presence of mouth breathing, a significant association is observed between the latter and rhinitis, as well as having increased *Y*-axis growth (standard dolichofacial growth), similar to what was observed by other authors.^13^^,^ 19 ^,^ 22 ^-^ 28 Surprisingly, there was no significant increase in the *Y*-axis growth when the patients were assessed for the presence of rhinitis. Perhaps the association between rhinitis and nasal obstruction, accompanied by mouth breathing, favors dental malocclusions (maxillary atresia, open bite, cross-bite, deep bite and dental crowding).^1^^,^ 12 ^-^ 14 ^,^ 18

It is worth mentioning that most patients with rhinitis assessed in this study did not have this condition diagnosed and among those with a medical diagnosis, few were adequately treated. Additionally, the fact that dental malocclusion was the reason why patients sought treatment at the service suggests that the symptoms of rhinitis were underestimated by the family and very often by the doctors who treated them. That shows the importance of a multidisciplinary assessment of patients with rhinitis and mouth breathing, to prevent complications such as dental malocclusion.

Another result obtained was a 36% prevalence of bruxism. This information reported by patients' parents may show low reliability; however, as the patients were young and had virtually no tooth wear from bruxism, it was the only way to get the information. Although prevalence rates of bruxism between 7% and 20% have been reported, rates of up to 60% have been documented, depending on the assessed population.^29^ Even though bruxism is reported as common among mouth breathers when compared to nasal breathers,^14^^,^ 30 this observation was not documented in this study. It is believed that bruxism occurs due to the need the individual has to equalize the pressures in the internal and external ear, since the mucosal edema caused by rhinitis extends to the mucosal lining of the Eustachian tube and, by causing its obstruction, it determines a pressure imbalance. The grinding of teeth would help balance the pressures.^18^^,^ 26

In conclusion, the frequency of rhinitis in children and adolescents undergoing orthodontic treatment is high; most of them have an allergic etiology associated with mouth breathing, which determines significant increase in the *Y* growth axis, clinically observed as dolichofacial growth tendency. A multidisciplinary approach of these patients is critical.
